# Development of a RVFV ELISA that can distinguish infected from vaccinated animals

**DOI:** 10.1186/1743-422X-6-125

**Published:** 2009-08-13

**Authors:** Anita K McElroy, César G Albariño, Stuart T Nichol

**Affiliations:** 1 Special Pathogens Branch, Division of Viral and Rickettsial Diseases, Centers for Disease Control and Prevention, Atlanta, GA 30333, USA; 2 Department of Pediatrics, Emory University, Atlanta, GA, USA

## Abstract

**Background:**

Rift Valley Fever Virus is a pathogen of humans and livestock that causes significant morbidity and mortality throughout Africa and the Middle East. A vaccine that would protect animals from disease would be very beneficial to the human population because prevention of the amplification cycle in livestock would greatly reduce the risk of human infection by preventing livestock epizootics. A mutant virus, constructed through the use of reverse genetics, is protective in laboratory animal models and thus shows promise as a potential vaccine. However, the ability to distinguish infected from vaccinated animals is important for vaccine acceptance by national and international authorities, given regulations restricting movement and export of infected animals.

**Results:**

In this study, we describe the development of a simple assay that can be used to distinguish naturally infected animals from ones that have been vaccinated with a mutant virus. We describe the cloning, expression and purification of two viral proteins, and the development of side by side ELISAs using the two viral proteins.

**Conclusion:**

A side by side ELISA can be used to differentiate infected from vaccinated animals. This assay can be done without the use of biocontainment facilities and has potential for use in both human and animal populations.

## Background

Rift Valley fever virus (RVFV) is a member of the family *Bunyaviridae *and as such is an enveloped virus that has a negative stranded RNA genome consisting of three fragments, aptly named S (small), M (medium) and L (large). The S segment codes for two proteins, a nucleocapsid protein that coats the viral genome in the virion, and a non-structural protein (NSs). The NSs protein is especially interesting, in that it is a filamentous nuclear protein[1], expressed by a virus that replicates and assembles in the cytoplasm of infected cells. The NSs protein is known to be involved in altering the host immune response because the virulence of viruses lacking a functional NSs is attenuated in mice, and these viruses are potent inducers of IFN α/β, unlike the wild type (WT) virus [2-4]. The M segment of the genome codes for two viral glycoproteins that are on the surface of the virion, as well as a nonstructural protein (NSm) that has unknown function. Finally, the L segment of the virus encodes the viral RNA polymerase.

RVFV is a mosquito-borne virus that causes significant morbidity and mortality in humans and livestock and is considered to be a bioterrorism threat agent. It was first identified in the 1930's in Kenya after isolation from a sheep in the Rift Valley [5]. It is present throughout Africa, and has also caused outbreaks in Madagascar off the Eastern coast of Africa as well as in Yemen and Saudi Arabia [6].

The virus is transmitted to humans by contact with infected livestock, usually through the butchering or the birthing process, or by the bite of an infected mosquito. Infected individuals typically have a mild disease consisting of fever, malaise, and myalgia; a very small percentage of these individuals will develop severe disease manifested as hepatitis, encephalitis, retinitis or hemorrhagic fever, which are the hallmarks of RVFV clinical disease. The overall fatality rate is estimated at 0.5-1%. However, in patients whose clinical illness is sufficiently severe to bring them to the attention of medical personnel, it has been reported to be as high as 29%, as was seen in the Kenya 2006-2007 outbreak [7].

RVFV is also a significant veterinary pathogen that affects livestock, such as cattle, goats, and sheep. Up to 90% mortality has been reported in newborn animals and as high as 30% in adult animals [8]. Consistent with its degree of pathogenicity in juvenile animals, RVFV is also extremely abortigenic; 40-100% of pregnant animals will abort during an outbreak [9]. Furthermore, livestock caretakers are exposed to virus in the process of caring for sick and dying animals, especially since amniotic fluid contains high quantities of virus.

There is a clear need for development of a safe efficacious vaccine to prevent these naturally occurring large scale outbreaks of severe disease in livestock and humans in the affected regions. The sporadic and explosive nature of these outbreaks makes vaccination control efforts challenging. It is very difficult in resource limited areas of Africa or the Middle East to sustain annual vaccination for a disease that appears infrequently. On the other hand, it is impossible to effectively vaccinate in the face of a rapidly moving ongoing epizootic. In addition, the regulatory hurdles and enormous expense to advancement of a human use vaccine make it unlikely that a product which targets poorly defined human populations in rural Africa and the Middle East would get developed. It has been observed that virus amplification cycles in livestock frequently precede human cases by 3-4 weeks, and play a critical role in the early stages of an outbreak. These highly viremic animals serve as an excellent source of direct contamination of humans, as well as a blood meal source for mosquitoes which can transmit the virus to humans. Recently, satellite derived data and rainfall measurements have proven to be effective predictors of time periods and geographical regions at high risk of experiencing RVF epizootics [10]. A viable strategy for control of RVF may be to use these predictive methods for targeted application of an inexpensive efficacious livestock vaccine which could prevent livestock epizootics, limit the vertebrate host virus amplification cycle and thereby also prevent human epidemics. Due to export restrictions and other regulatory issues, acceptance of such a vaccine would require development of a companion diagnostic assay that could differentiate between infected and vaccinated animals (DIVA).

There is currently no licensed vaccine available for use in the US or Europe and vaccine options in Africa and the Middle East are limited. A formalin inactivated RVFV vaccine has limited availability in the US for protection of military personnel and laboratory workers [11-17]. Two live attenuated viruses have been tested in various animals as potential vaccine strains. A mutagen-attenuated strain (MP12) and the live attenuated Smithburn strain have been tested in pregnant ewes and lambs, as well as in pregnant, fetal, neonatal and adult bovids. The results of these studies with live vaccines are varied, in some instances showing no clinical illness and the development of neutralizing antibody titers as well as protection from challenge [18-21], and in other studies showing the viruses to be abortogenic and teratogenic [22,23]. Therefore neither of these virus strains appears to be an ideal candidate for a vaccine strain because of their questionable safety profiles, in addition to their lack of DIVA capability.

In recent years, a reverse genetics system has become available for RVFV, thereby facilitating studies of viral pathogenesis and the development of specifically attenuated vaccine strains [24,25]. This system has been used to generate viruses that are missing the NSs protein, the NSm protein, or both. These live attenuated vaccine candidates provide complete protection with a single administration in the highly sensitive Wistar-Furth rat model [26]. ΔNSm/ΔNSs virus infected rats demonstrate a strong antibody response to the N protein, but as expected, no antibody response to the NSs protein. In contrast, rats infected with WT virus demonstrate an antibody response to both the N and NSs proteins by immunofluorescence analysis. The ΔNSm/ΔNSs virus has immense potential as a vaccine for use in the model proposed above where predictive methods guide targeted vaccine strategies to prevent livestock epizootics. Not only is the exact genetic makeup of this virus known, since it was generated from cloned cDNA, but it is more attenuated than the currently available attenuated strains, MP12 and Smithburn. The ΔNSm/ΔNSs virus bypasses the problem of possible reversion to virulence by having two large deletions, one on the M segment and one on the S segment of the genome. In addition, unlike the currently available attenuated strains, the ΔNSm/ΔNSs vaccine meets the DIVA requirement by virtue of the missing NSs protein.

In this study, we build upon the observation that infection with the mutant virus can be distinguished from infection with the WT virus by immunofluorescence analysis. We describe the generation of an ELISA that can distinguish infected from vaccinated animals. This companion assay can easily be performed in a rudimentary laboratory setting and would be ideal for use the in resource poor countries where RVFV is prevalent.

## Results and Discussion

### Cloning, expression and purification of RVFV N and NSs

ELISAs have been used in the past in the diagnosis of RVFV infection in both humans and livestock [27-31], and these assays have either used whole cell lysate derived from infected cells [32] or purified N protein as antigen. Two viral proteins, N and NSs would be required in order to develop an ELISA that could distinguish vaccinated from infected animals. The ORF's of RVFV N and NSs from strain ZH501 were amplified by PCR and cloned into the pET20(+)b expression vector with the goal of achieving soluble expression of His-tagged versions of the proteins in bacteria. The pET20(+)b vector has a signal sequence at the N-terminus that directs the expressed protein to the periplasmic space which should promote folding and disulfide bond formation and theoretically enhance solubility. However, despite multiple attempts using protocols for purification of native protein, an appreciable amount of neither soluble N nor NSs protein were able to be purified (data not shown).

**Figure 1:**
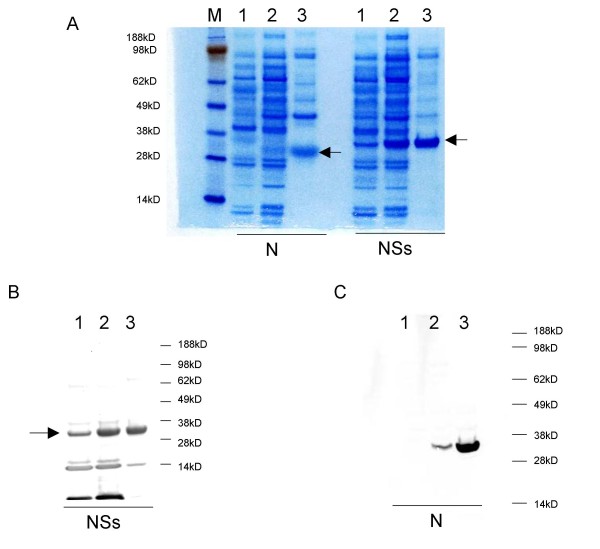
**Expression and purification of the RVFV N and NSs antigens**. Protein samples were mixed with reducing sample buffer and run on SDS PAGE gels as described in Methods. (A) Induction and purification of N and NSs. M: molecular weight maker. 1: uninduced whole cell lysate from *E. coli *that was transformed with a plasmid that expressed either the RVFV N or NSs protein, Lane 2: whole cell lysate from the samples after induction with IPTG, Lane 3: purified N or NSs protein. Gels were transferred to PVDF membranes and western blotted for either the NSs protein (1B) or the N protein (1C) with human polyclonal or mouse monoclonal sera respectively. On each gel lanes 1, 2, and 3 represent uninduced whole cell lysate, induced whole cell lysate and purified protein.

Successful induction was readily achieved for both the N and NSs proteins by IPTG induction (Figure 1A). Use of a denaturing protocol (as described in Methods) for purification of His-tagged N and NSs was successful in purifying the respective proteins (Figure 1). The N and NSs proteins both eluted most efficiently in the first and second elutions with Bfr E (data not shown, see Methods). Confirmation of the identity of the expressed proteins was made by western blotting using antibodies specific for the respective protein (Figure 1B and 1C). The induction of N was very tightly controlled, but as indicated by lane 1 of Figure 1B, there was some leaky expression of NSs prior to induction.

### Titration of antigens

**Figure 2:**
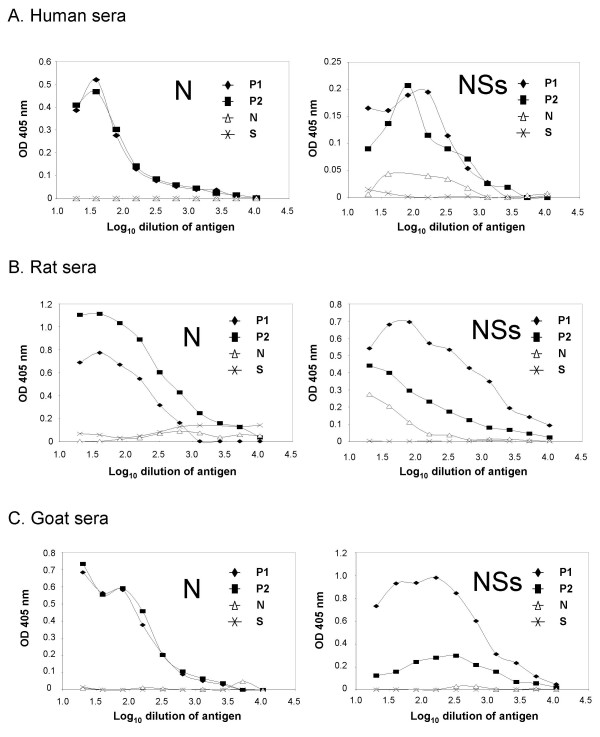
**Titration of antigens with various sera**. Antigens were serially diluted and coated onto EIA plates. After overnight binding and then blocking, the plates were incubated with a 1:100 dilution of human (A), rat (B) or goat (C) sera and the appropriate secondary antibody as described in materials and methods. P is a positive control serum, N is a negative control serum and S is a secondary alone control.

The N and NSs antigens were serially diluted in PBS and coated onto EIA plates. A negative control bacterial cell lysate that had been run through the same purification protocol was run in parallel with the N and NSs antigens and the negative lysate OD values were subtracted from the experimental sample OD values prior to analysis in order to control for non-specific binding. Two positive control sera from each of the tested species (goat, rat and human) were used to determine the optimal amount of protein to use in the assay. The antigen titration curves for N and NSs demonstrated linearity at 200 ng/well (corresponding to the part of the curve between 2.0 and 2.5 logs) (Figure 2A, B, C) for all three species, therefore this was chosen as the concentration to be used in all further assays. Species specific negative control sera confirmed the specificity of the assay and secondary only controls demonstrated the low level of background in these assays.

### Analysis of the antibody response in rats and demonstration that ELISA can be effectively used to distinguish animals infected with wt RVFV from those vaccinated with a ΔNSs virus

**Figure 3:**
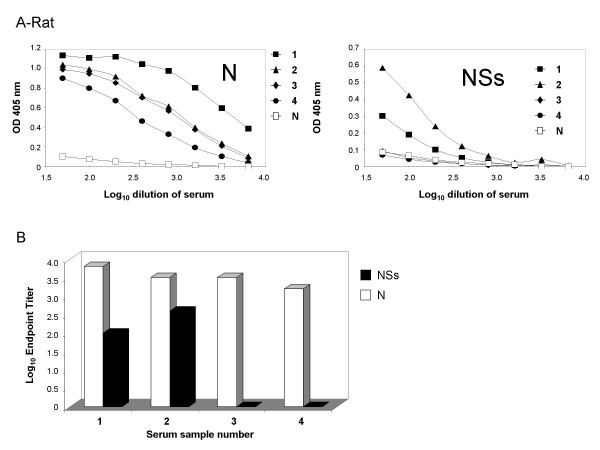
**Comparison of the N and NSs response in various rat sera**. Antigens were coated onto EIA plates as described in Methods. After overnight binding and then blocking, the plates were incubated with serially diluted rat sera, and then with anti-rat HRP. Samples 1 and 2 are from rats that were infected with WT RVFV; samples 3 and 4 are from rats that were infected with the ΔNSs virus. Sample N is a negative control rat sera. Figure A demonstrates the dilution curves for each sample. Figure B demonstrates the endpoint titers for each antigen for the positive samples.

Four representative rat sera were tested against the two experimental antigens. These sera were obtained from rats that had been infected with WT RVFV (samples 1 and 2), or vaccinated with a ΔNSs virus (samples 3 and 4) [26]. Sera from all animals demonstrated the expected dose response curves (Figure 3A). As was expected, animals that were vaccinated with the virus that was missing the NSs protein did not have an antibody response to the NSs antigen. Therefore these side by side ELISAs were effective at distinguishing infected from vaccinated animals. These data were also used to calculate endpoint titers for each animal that was tested (Figure 3B). The endpoint titer is the log of the sample sera dilution at which the signal remains at least two-fold above that of the negative sera control. The endpoint titer provides a way to normalize between assays that test sera from different species since there are varying degrees of background and raw signal based upon the species being tested. Serum samples from WT infected rats in general had lower antibody responses to NSs (as determined by endpoint titers) than to the N antigen. This phenomenon was also observed with the other two species as is described below.

### Antibody response in goats

**Figure 4:**
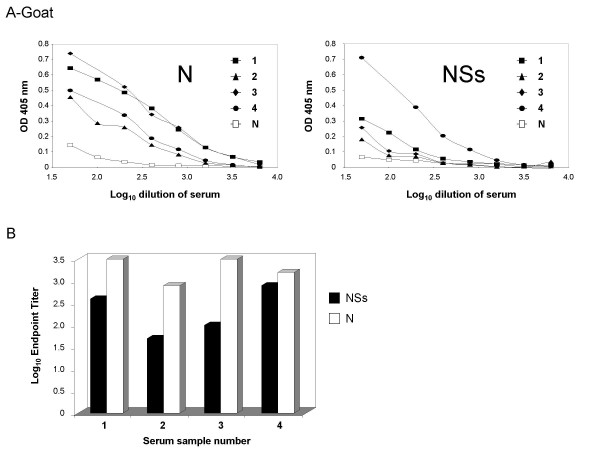
**Comparison of the N and NSs response in various goat sera**. Antigens were coated onto EIA plates as described in Methods. After overnight binding and then blocking, the plates were incubated with serially diluted goat sera, and then with anti-goat HRP. Samples 1 through 4 are from naturally infected goats. Sample N is a negative control goat sera. Figure A demonstrates the dilution curves for each sample. Figure B demonstrates the endpoint titers for each antigen for the positive samples.

In an effort to demonstrate the utility of the assay in a naturally occurring animal host, four representative goat sera that were obtained from the Jizan province in Saudi Arabia during the RVFV outbreak in 2000 were tested for antibody response to N and NSs. Sera from all animals demonstrated the expected dose response curves (Figure 4A). These data were also used to calculate endpoint titers for each animal that was tested (Figure 4B). The endpoint titers for goat sera were similar to those for the rat sera that were tested. Three of the four animals had a signficantly greater antibody response to the N protein than to the NSs protein, which was also observed in the assays done with rat sera, however all four goats had an antibody response against both antigens.

This assay would therefore be useful in the diagnosis of RVFV infection in goats and could be used to distinguish animals that had been infected with WT virus from animals that had been vaccinated with the ΔNSs vaccine strain. Safety and efficacy studies using the ΔNSs vaccine strain will be initiated in livestock species in the near future which will allow generation of additional specimens to further characterize the specificity and dynamics of the N and NSs ELISAs.

### Antibody response in humans

**Figure 5:**
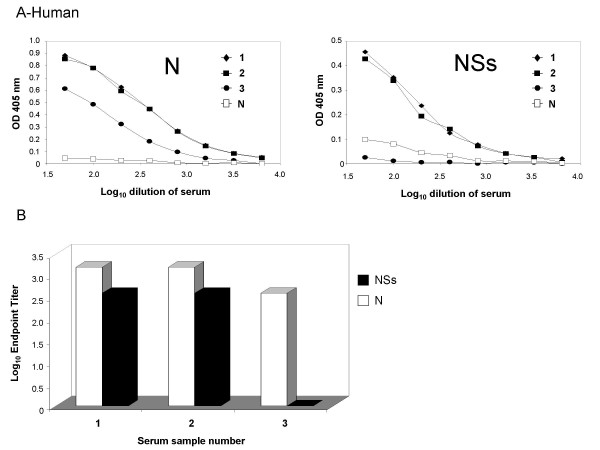
**Comparison of the N and NSs response in various human sera**. Antigens were coated onto EIA plates as described in Methods. After overnight binding and then blocking, the plates were incubated with serially diluted human sera, and then with anti-human HRP. Samples 1 and 2 are from naturally infected humans. Sample 3 is from a human that was vaccinated with inactivated WT RVFV, and sample N is a negative control human sera. Figure A demonstrates the dilution curves for each sample. Figure B demonstrates the endpoint titers for each antigen for the positive samples.

Representative human sera were tested to determine the level of antibody response to the two antigens. Samples 1 and 2 were obtained from naturally occurring RVFV infections. Sample 3 was from an individual who had been vaccinated with inactivated RVFV. All human sera that were used are part of the Special Pathogens Branch reference collection. All dose response curves demonstrated the expected progressive slope (Figure 5A). It is interesting to note that in the naturally occurring infection, an antibody response against both N and NSs was detected; however, in the vaccinated individual there was only an antibody response to the N protein. All samples had a similar level of antibody response to the N protein as indicated by the endpoint titers (Figure 5B), and these were comparable to those observed for rats and goats. The lack of response of the vaccinated individual to the NSs protein was expected since viral gene expression is required for the production of the NSs protein, and this individual was vaccinated with an inactivated virus.

**Figure 6:**
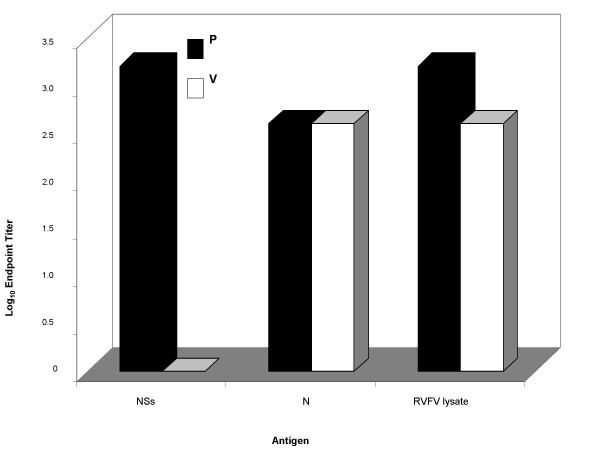
**N and NSs derived assays are comparable to the current gold standard assay using RVFV infected cell lysate**. RVFV infected cell lysate at 1:2000 dilution or N or NSs at 200 ng/well were coated onto EIA plates and allowed to absorb overnight. The assay was carried out as described in Methods. Endpoint titers against each antigen from a human case that was naturally infected (P) and from a human that was vaccinated (V) are shown.

This assay could prove to be useful in the diagnosis of human disease especially since it can be easily replicated without the need for a special containment laboratory to produce antigen. This protein based ELISA would be much more accessible to researchers and clinicians who work in regions of the world where this virus is prevalent. To demonstrate this point, we compared the assay that is currently being used for diagnosis at the CDC's Disease Assessment Group of the Special Pathogens Branch [32] with our assay using human sera (Figure 6). As is demonstrated using the method of endpoint titers, either antigen produced comparable results, therefore the N or NSs based assays would be equally effective at diagnosis, but would not require BSL-4 for antigen production.

## Conclusion

RVFV causes morbidity and mortality in humans and livestock that leads to major social and economic consequences in the developing world. The virus is always present at endemic levels in the population; however, during periods in which human epidemics arise, it has been observed that they are preceded by epizootics in livestock. These livestock epizootics serve as an amplification step in the spread of the virus. Prevention of disease in animals through the use of a safe and effective vaccine would not only protect livestock, upon which humans depend for both survival and their livelihood, but it would also serve to prevent human disease by breaking the amplification cycle.

Recent studies done by Bird et al have demonstrated that a virus can be created using reverse genetics that is missing one or more viral virulence factors. These viruses are completely apathogenic in rats and able to provide 100% protection from challenge with WT virus. The data presented in this paper expands upon those earlier studies to provide an easily accessible assay that can be reliably used to distinguish animals that are infected with WT virus from animals that have been vaccinated. This differential ability is important for vaccine acceptance given regulations restricting movement and export of infected animals in the affected areas. In addition to indirectly reducing human morbidity and mortality through the decrease in epizootics, livestock vaccination would also assist rural human populations by protecting one of their most valuable economic resources.

## Methods

### Cloning of N and NSs genes

PCR was used to amplify the open reading frame of N and NSs from the pCAGGS N and NSs vectors respectively [26]. Primers used for N were as follows: RVFV S Hind III 5' CGA AGC TTG ACA ACT ATC AAG AGC TTG 3'and RVFV S XhoI 5' CGC TCG AGG GCT GCT GTC TTG TAA GCC 3'. Primers used for NSs were as follows: RVFV NSs Hind III 5' CGA ACG TTG ATT ACT TTC CTG TGA TAT C 3' and RVFV NSs XhoI 5' cgc tcg aga tca acc tca aca aat cca tc 3'. PCR reactions contained 1× AccuPrime Buffer I (Invitrogen), 10 ng plasmid template, 200 nM of each primer, and 1 ul of AccuPrime Taq DNA Polymerase (Invitrogen). The following parameters were used for PCR: 94°C for 2 min, then 35 cycles of 94°C for 30 sec, 56°C for 30 sec and 68°C for 1 min with a final extension of 7 min at 68°C. PCR products were verified by gel electrophoresis and then prepared for restriction digest using the QIAquick PCR purificaton kit (Qiagen). PCR products and target vector pET20(+)b (Novagen) were digested with Xho I and Hind III in NEB Buffer #2. Digested products were gel purified, and then ligation of pET20(+)b vector with each of N and NSs were performed overnight using T4 DNA ligase in 1× ligase buffer (NEB) at 16°C. Ligations were transformed into competent TOP10 *E. coli *(Invitrogen) and plated onto LB with 100 ug/ml ampicillin. Plates were incubated overnight at 37°C and colonies were selected for analysis. After overnight growth in liquid culture and miniprep purification, the plasmids were analyzed by restriction digest with EcoRI to verify correct insertion. pET20(+)bRVFV NSs cut with EcoRI was expected to have products of 212 and 4280 bp and pET20(+)bRVFV N cut with EcoRI was expected to have products of 668 and 3771 bp. Clones with the correct restriction digest pattern were sequenced using standard techniques to verify gene sequence as well as the presence of the His-tag at the C-terminus of the complete open reading frame for each protein.

### Purification of RVFV N and NSs proteins

pET20(+)bRVFV NSs, pET20(+)bRVFV N or pET20(+) (empty vector) were transformed into competent BL21 (DE3) *E. coli *(Novagen) and an isolated colony of each was selected and grown in liquid LB with 100 ug/ml ampicillin until OD600 was between 0.6 and 1.0 then cultures were stored overnight at 4°C. The following morning, cultures were pelleted for 5 min at 5000 × g. Pelleted bacteria were resuspended in 10 mL LB medium with 100 ug/ml ampicillin and innocuated into 500 ml LB with 100 ug/ml ampicillin. Cultures were incubated at 37°C while shaking until OD600 was 0.6, then expression was induced by adding IPTG to a final concentration of 0.6 mM and cultures were grown at 37°C for an additional 4 hours. Bacteria were pelleted for 10 min at 10,000 × g and stored at -70°C.

Bacterial pellets were thawed and lysed in 5 ml of Buffer B (8 M urea, 0.1 M sodium phosphate buffer, 0.01 M Tris-Cl, pH 8.0) per gram of pellet with the addition of protease inhibitors (Roche). Lysate was incubated at RT for 1 hour with rocking. Lysate was cleared by centrifugation at 10,000 × g for 30 min at room temperature. Supernate was stored at -70°C.

Batch purification of His-tagged proteins was achieved by incubation of 4 ml of cleared lysate with 1 ml of 50% slurry Ni-NTA His·Bind Resin (Novagen) with rocking at room temperature for 1 hour. Mix was allowed to settle in a chromatography column and flow through was collected. Column was washed twice with 4 ml of Buffer C (8 M urea, 0.1 M sodium phosphate buffer, 0.01 M Tris-Cl, pH 6.3). Elution with Buffers D (8 M urea, 0.1 M sodium phosphate buffer, 0.01 M Tris-Cl, pH 5.9) and E (8 M urea, 0.1 M sodium phosphate buffer, 0.01 M Tris-Cl, pH 4.5) were each performed four times with 1 ml of the respective buffer.

Samples were analyzed on 4-12% Bis-Tris gels which were stained with Simply Blue Safe Stain (Invitrogen).

### Western Blotting

Purified fractions of N and NSs were run on 12% Bis-Tris gels in 1× MES buffer per manufacturer's instructions (Invitrogen). Gels were transferred to PVDF membranes using the iBlot Gel Transfer Device (Invitrogen). Blots were blocked in blocking buffer (5% skim milk in TBS with 0.1% tween 20) for 1 hour at RT. The blots were then placed in primary antibody diluted in blocking buffer and incubated for 1 hour at RT. Mouse monoclonal against the N protein was used at 1:500 and was generated by the Special Pathogens Branch, and human polyclonal was used at 1:1000 and is a reference sample from the Special Pathogens Branch. Blots were washed in TBST (1× TBS with 0.1% tween 20) 3 times for 5 min each then placed in secondary antibody; goat anti-mouse HRP (KPL) or goat anti-human HRP (Jackson ImmunoResearch) diluted 1:20,000 in blocking buffer for 1 hour at RT. Blots were again washed 3 times in TBST for 5 min each. Blots were placed in Supersignal West Dura Reagent (Pierce) for 5 min and signal was detected on an Alpha Innotech FluroChemHD2 imager.

### Enzyme linked immunosorbant assay

Purified N, purified NSs, negative control bacterial cell lysate, whole cell lysate from RVFV infected Vero E6 cells, or negative control cell lysate from uninfected Vero E6 cells were diluted in PBS and allowed to absorb overnight onto 96 well EIA plates (Costar). N and NSs antigens were applied to EIA plates either in serial dilutions for antigen titration experiments, or at a concentration of 200 ng/well for serum dilution experiments. Negative control bacterial cell lysate was applied to a separate plate at an equivalent volume. Whole cell lysates from RVFV infected Vero E6 cells or uninfected Vero E6 cells were used at 1:2000 per established diagnostic protocols. Plates were blocked in 1× blocking buffer (5% skim milk, 5% fetal bovine serum, and 0.1% tween 20 in 1× PBS) at 37°C for 1 hour. Plates were then incubated with primary antibodies at specified dilutions in blocking buffer for 1 hour at 37°C. Plates were washed 3 times in PBST (1× PBS with 0.1% tween 20) and then incubated with goat anti-rat HRP (1:10,000), bovine anti-goat HRP (1:10,000), or goat anti-human HRP (1:10,000) (Jackson ImmunoResearch), diluted in blocking buffer for 1 hour at 37°C. Plates were washed 3 times in PBST prior to the addition of ABTS substrate used according to the manufacturer's instructions. Reactions were stopped with the addition of 1% SDS and read at 405 nM. All samples were run in duplicate and averages were used in the analysis. Absolute values obtained from negative control lysates were subtracted from values obtained from the experimental antigen prior to analysis to control for non-specific binding.

## Abbreviations

The following abbreviations were used in the manuscript: RVFV: Rift Valley Fever Virus; ELISA: Enzyme Linked Immunosorbant Assay; EIA: Enzyme Immuno Assay; PBS: Phosphate Buffered Saline; HRP: Horseradish Peroxidase; TBS: TRIS Buffered Saline; and DIVA: Differentiate between Infected and Vaccinated Animals.

## Competing interests

The authors declare that they have no competing interests.

## Authors' information

Anita K. McElroy is a resident in the Department of Pediatrics at Emory University. She is a participant in the American Board of Pediatrics Integrated Research Pathway. This work was performed while she was a recipient of the NIH loan repayment program award.

## Authors' contributions

CA assisted in the design of the study and the molecular cloning. AKM performed the cloning, gene expression, purification, immunoassays and drafted the manuscript. STN conceived of the study and participated in its design and coordination. All authors read and approved of the final manuscript.
